# Gradients not needed: ML-driven propagation of nonadiabatic molecular dynamics without reference gradients

**DOI:** 10.1039/d5sc09557c

**Published:** 2026-01-22

**Authors:** Mikołaj Martyka, Joanna Jankowska, Hans Lischka, Pavlo O. Dral

**Affiliations:** a University of Warsaw, Faculty of Chemistry 02-093 Warsaw Poland jjankowska@chem.uw.edu.pl; b Department of Chemistry and Biochemistry, Texas Tech University Lubbock TX USA hans.lischka@ttu.edu; c State Key Laboratory of Physical Chemistry of Solid Surfaces, College of Chemistry and Chemical Engineering, Fujian Provincial Key Laboratory of Theoretical and Computational Chemistry, Xiamen University Xiamen Fujian 361005 China dral@xmu.edu.cn; d Institute of Physics, Faculty of Physics, Astronomy, and Informatics, Nicolaus Copernicus University in Toruń Grudziadzka 5 87-100 Toruń Poland; e Aitomistic Shenzhen 518000 China

## Abstract

The recent development of machine learning (ML) methods for quantum chemistry has tremendously boosted the efficiency of molecular calculations. In this work, we use ML to enable nonadiabatic molecular dynamics (NAMD) simulations without access to the analytical energy gradients from the underlying target electronic structure method. By fine-tuning our foundational model for excited states, OMNI-P2x, on energies alone and leveraging automatic differentiability to obtain forces, we eliminate the gradient computation bottleneck that restricts calculations, such as NAMD, to methods with available analytical derivatives. First, we validate the method on the benchmark system, fulvene, demonstrating that gradient-free ML potentials accurately reproduce NAMD populations and dynamics across multiple levels of theory: AIQM1/MRCI, CASSCF, and MRSF-TDDFT. This enables, for the first time, performing dynamics at the QD-NEVPT2 level, where analytical gradients remain unavailable. We further benchmark the protocol on cyclohexadiene photoinduced ring-opening, where gradient-free training on XMS-CASPT2 energies reproduces reference dynamics with high accuracy, and compare them to QD-NEVPT2 results. Finally, we apply the approach to *trans*-azobenzene, a prototypical molecular photoswitch, by performing fully dimensional simulations of its photoisomerization dynamics at the CASSCF and QD-NEVPT2 levels, establishing the highest-level excited-state simulations of this photoreaction to date.

## Abstract 2

ML enables nonadiabatic molecular dynamics simulations without access to the analytical energy gradients from the underlying target electronic structure method.

## Graphical abstract

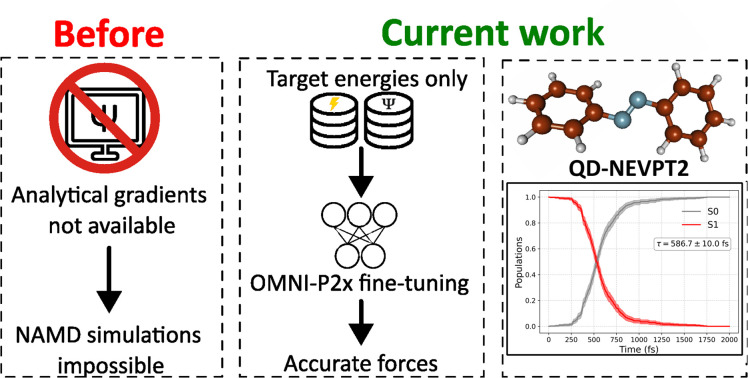

## Introduction

Nonadiabatic molecular dynamics (NAMD) simulations are a powerful tool in computational photochemistry, enabling the real-time study of complex photoprocesses and providing insights that static calculations cannot capture. Among dynamic simulation approaches, NAMD uniquely allows the direct prediction of experimentally relevant quantities, such as photoswitching quantum yields, excited-state lifetimes, and time-dependent multi-photon effects. The most widely used technique within this framework is trajectory surface hopping (TSH),^1^ a mixed quantum-classical method successfully applied to a broad range of photoresponsive systems. TSH represents the fully quantum molecular wavepacket as a swarm of independent semiclassical trajectories, which can “hop” between adiabatic potential energy surfaces (PESs). This methodology has allowed researchers to study important, photochemical and photophysical processes, such as operation of photoswitches,^2,3^ photodegradation mechanisms of amino acids^4^ and DNA nucleobases,^5^ molecular systems for clean energy generation,^6,7^ as well as photoinduced process relevant to organic synthesis.^8,9^

At the same time, NAMD simulations are extremely computationally demanding, as even a modest set of trajectories for ultrafast photoprocesses requires hundreds of thousands of energy and gradient evaluations, thereby restricting highly accurate methods to small model systems. Computing energy gradients constitutes a major bottleneck in both NAMD propagation and ML training data generation. For example, in the mixed-reference spin-flip TD-DFT^10–12^ dynamics shown later in the manuscript, computing the energy gradients accounts for approximately 80% of the CPU time associated with a single-point calculation. This bottleneck becomes far more severe for methods without analytical gradient implementations—a critical limitation for photochemistry, as many highly accurate multireference methods such as QD-NEVPT2 (ref. 13 and 14) and certain CASPT2 (ref. 15) variants lack available analytical gradients. For these methods, gradients must be obtained through numerical differentiation, requiring single-point energy calculations at a large number of perturbed geometries, effectively blocking access to state-of-the-art quantum chemical methods.

Machine learning interatomic potentials (MLIPs) provide a convenient way of procuring expensive energy gradients, due to their differentiability, which is required for gradient backpropagation^16^ in the training process. This implies that any MLIP that can provide predictions of molecular energies can also provide energy gradients, usually with very low overhead due to the efficiency of the autograd procedure.^17^ Despite this capability, nearly all MLIP training protocols incorporate reference gradients into the loss function, as fitting derivatives alongside energies substantially improves accuracy.^18–22^ Hence, the challenge lies not in obtaining ML gradients, but in training models that provide dynamics-quality forces without access to the energy gradients of the underlying target level of theory.

For excited-state simulations the challenge of gradient-free training is even more formidable due to the increased complexity of fitting multiple, interacting potential energy surfaces. Indeed, nearly all reported studies that employed MLIPs to accelerate NAMD did so by training on gradients, which has become the current state of the art.^23–31^ The absence of analytical gradients in the reference method is de-facto an exclusion criterion for the choice of such a method for attempting MLIP-accelerated NAMD. Only a few early studies performed gradient-free training either due to limitations in the implementations not supporting training on gradients or for benchmark purposes^22,32,33^ documenting the requirement for a big number of reference energies to achieve sufficient quality. The challenge for data-efficient gradient-free training of MLIPs for NAMD remains open.

Here, we propose a solution to this challenge that is based on the recent advances in fine-tuning of pre-trained models. Inspiring examples are models for the ground state trained on multi-fidelity data, where the highest fidelity only provided energies.^34–36^ Other examples are all-in-one^37^ and multi-task learning strategies^38^ that enable training on several data sets simultaneously. While no such examples were reported for excited states yet, related research^30,39,40^ demonstrates that transfer learning—particularly fine-tuning from pre-trained foundational models—can dramatically enhance data efficiency when trained on energies and gradients, with an over 10× increase reported in ref. 40. This unprecedented efficiency raises a compelling question: if high accuracy can be achieved with minimal training data, can we eliminate the need for the high-level gradient computation bottleneck entirely by training only on energies of the target level of theory?

In this work, we demonstrate that fine-tuning the foundational OMNI-P2x model^40^ on energies alone enables data-efficient accurate NAMD simulations without reference gradients, relying only on differentiating an MLIP trained on energies. This unlocks dynamics at previously inaccessible levels of theory—most notably QD-NEVPT2—where analytical gradients remain unavailable. We validate the approach across three systems of increasing complexity: fulvene (a common NAMD benchmark), cyclohexadiene (a fundamental photoinduced ring-opening system), and *trans*-azobenzene (a prototypical photoswitch). For azobenzene, we perform the first fully dimensional simulations at both CASSCF and QD-NEVPT2 levels, establishing the highest-level excited-state description of this photoreaction to date and overcoming limitations that have restricted prior studies to lower levels of theory^41–44^ or reduced dimensionality.^45,46^

## Results

### Nonadiabatic dynamics of fulvene

To assess the performance of gradient-free propagation of nonadiabatic molecular dynamics, we start with the example of fulvene, which is a popular benchmark molecule for NAMD studies. It has been selected as a molecular representation of Tully's model III^47,48^ and has been used to benchmark both ML models^29,30,49^ and novel quantum chemical methods and algorithms for trajectory surface hopping.^50–52^

**Fig. 1:**
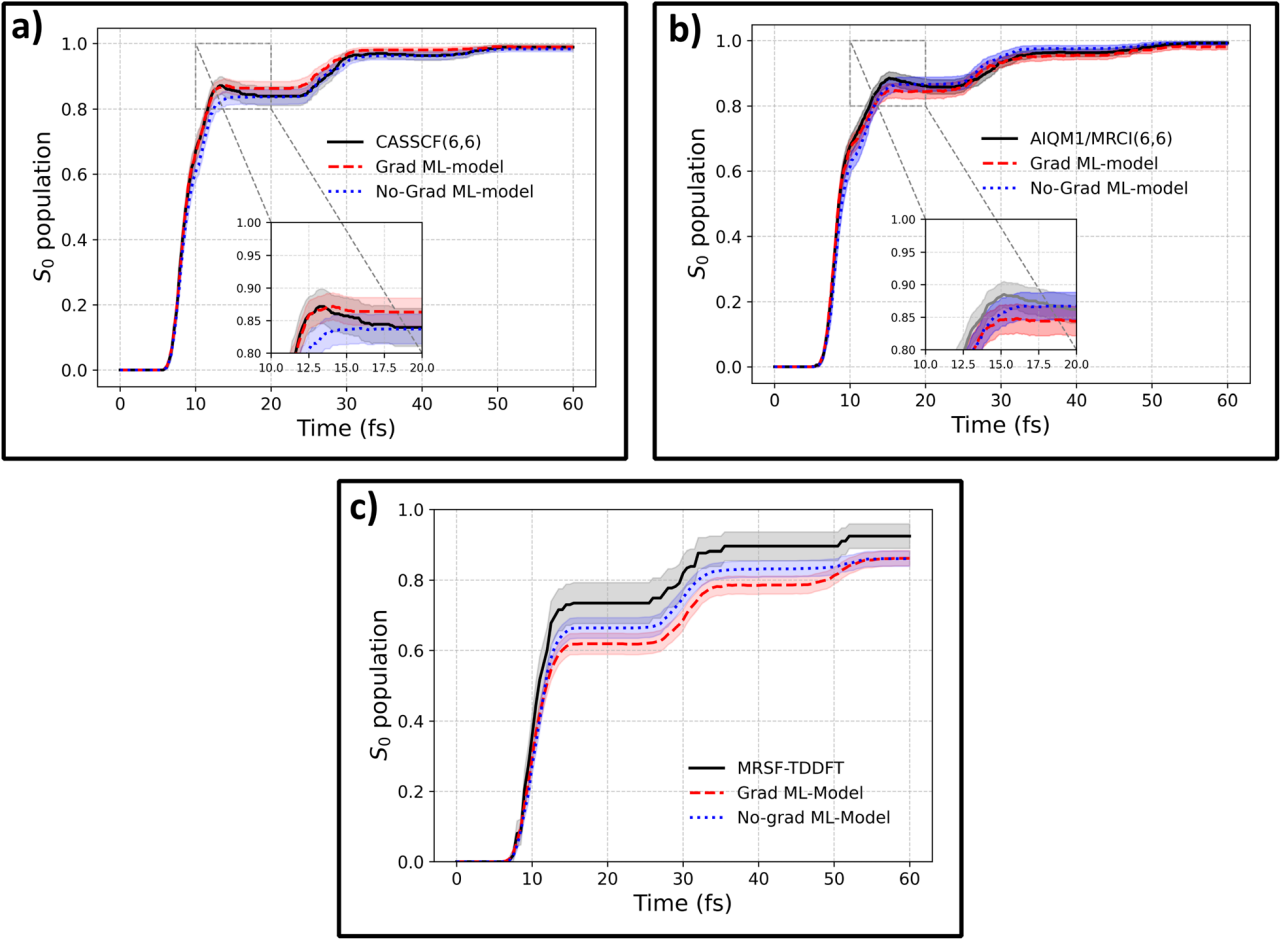
Ground-state population recovery in fulvene following S_0_ → S_1_ excitation, demonstrating that gradient-free ML models reproduce reference dynamics as accurately as models trained with gradients. ML models were fine-tuned from OMNI-P2x on datasets at three levels of theory: (a) CASSCF(6,6), (b) AIQM1/MRCI(6,6), and (c) MRSF-TDDFT. Black solid lines show reference quantum-chemical dynamics, red dashed lines show ML models trained with energies and gradients, and blue dotted lines show gradient-free models trained on energies alone. Shaded regions represent 95% confidence interval error bars. The S_1_ population has been omitted from the plot, as it follows the relationship *P*(S_1_) = 1 − *P*(S_0_).

The training dataset containing 3550 fulvene configurations was taken from ref. 40, where it was collected using an active transfer learning (AL) procedure targeting the CASSCF(6,6) level of theory, using the OMNI-P2x model as a starting point. Ref. 40 only reported the results for the standard protocol of propagating NAMD with a MLIP trained on both energies and gradients. Here, we extend this study by testing whether fine-tuning only on energies can enable reliable NAMD (see the Methods for details). Importantly, electronic state populations obtained with a MLIP trained only on CASCF(6,6) energies agree excellently with the reference CASSCF(6,6) dynamics (Fig. 1a). Moreover, there is no statistically significant difference between the gradient-free model and the model trained with gradients within a 95% confidence interval.

To ensure that the accuracy of the gradient-free training is not a consequence of the level of theory used in the AL procedure, we re-label all training data points with two different levels of theory: AIQM1/MRCI-SD(6,6), where excitations are calculated using a semi-empirical quantum mechanical Hamiltonian, and mixed-reference spin-flip TD-DFT (MRSF-TDDFT). Both gradients and energies were computed using the reference methods, and two sets of MLIPs were trained: one without access to the reference gradients (No-Grad ML-Model) and another with access to the energy gradients (Grad ML-model), which served as a comparison.

To improve data efficiency, the training process used an all-in-one learning strategy.^37^ Aside from the target level of theory (MRSF-TDDFT or AIQM1/MRCI), fine-tuning of the foundational OMNI-P2x model was also performed on energies and gradients from the original CASSCF(6,6)-level dataset, which served as an auxiliary level of theory. This dataset was sampled from our previous active learning;^40^ we re-labeled each molecular conformation in the dataset with the aforementioned levels of theory. The Grad and No-Grad ML models differed in having or not having access to energy gradients at the targeted level of theory. The level of theory was one-hot-encoded, as described in ref. 37 and 40.

The MLIPs fine-tuned on data with and without target energy gradients are used to propagate NAMD trajectories at the target level, with the electronic state populations presented in Fig. 1b and c for both methods. In the case of AIQM1, the agreement with the reference is outstanding for both MLIPs. For the MRSF-TDDFT dynamics, the agreement between the ML-predicted and reference state populations is slightly reduced compared to the other methods, yet it remains acceptable, with an average relative error of 9.3% in the S_1_ population. Importantly, no statistically significant differences are observed between the population dynamics obtained with ML models trained with or without target-level gradients. While the agreement could likely be further improved by sampling additional points specific to this level of theory, the current results remain within the 95% confidence interval for the gradient-free model over most of the trajectories. It should be noted that MRSF-TDDFT calculations exhibited numerical stability issues (see the Methods), with only 55% of trajectories completing successfully, introducing additional intrinsic uncertainty in the reference populations. A discussion about the effects of incompleteness of reference data on the agreement with ML-NAMD simulations can be found in Section S1 of the SI. Importantly, if bootstrapping with resampling reduced to 55% of trajectories per iteration is used, the differences between the reference and ML population curves become statistically insignificant.

Having shown that by fine-tuning OMNI-P2x on data without target-level energy gradients we can accurately simulate NAMD, we now leverage this protocol to perform NAMD simulations of fulvene at the QD-NEVPT2 level of theory. Due to the lack of available analytic gradients, this would be an impossible task using the reference methods directly, as running 1000 trajectories with numerical gradients would require over 4 million CPU-hours, corresponding to 6N single-point calculations per gradient evaluation (*N* = 12 atoms), and 1000 trajectories propagated for 60 fs at a 0.1 fs time step. Using our ML procedure, this task took below 400 CPU hours to label the data and around 20 minutes on a 16-core node to propagate the final 1000 trajectories.

**Fig. 2:**
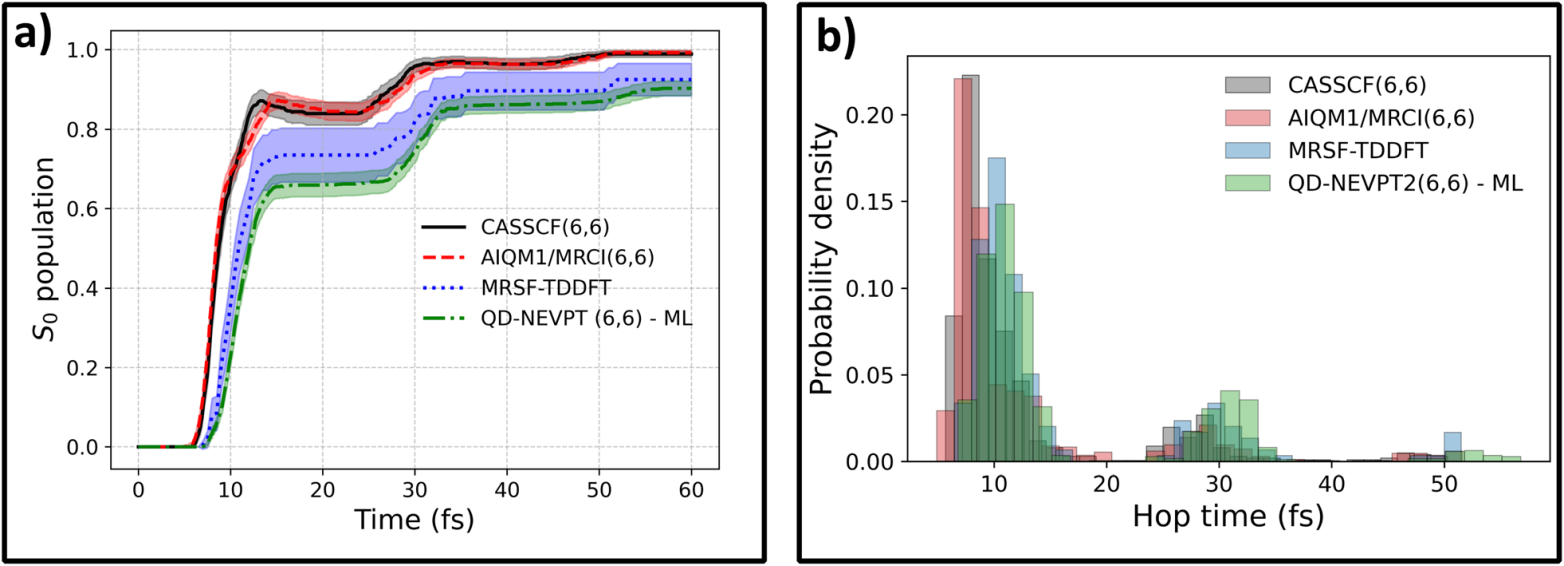
Comparison of nonadiabatic dynamics in fulvene following S_0_ → S_1_ excitation, propagated at four levels of theory. Panel (a) shows the ground-state population rise. ML@QD-NEVPT2 (green, dot-dashed) is compared to CASSCF(6,6) (black, solid), MRSF-TDDFT (blue, dotted) and AIQM1/MRCI(6,6) (red, dashed) populations obtained with reference methods with analytical gradients. Panel (b) shows the distribution of S_1_ → S_0_ hopping times for all four levels of theory. Shaded regions represent 95% confidence interval error bars.

Now we can turn to a comparison of ML-enabled QD-NEVPT2 dynamics with the dynamics at other levels of theory with available reference energy gradients. Interestingly, the ML@QD-NEVPT2 population differs significantly from both the CASSCF and AIQM1/MRCI results and more closely resembles the MRSF-TDDFT curve (Fig. 2a). This is also evidenced in the hopping time distribution: both MRSF-TDDFT and ML@QD-NEVPT2 predict a significantly larger fraction of the population to deactivate in the second wave of hopping, eventually resulting in a slower relaxation to the ground state (Fig. 2b).

### Photoinduced ring opening in cyclohexadiene

**Fig. 3:**
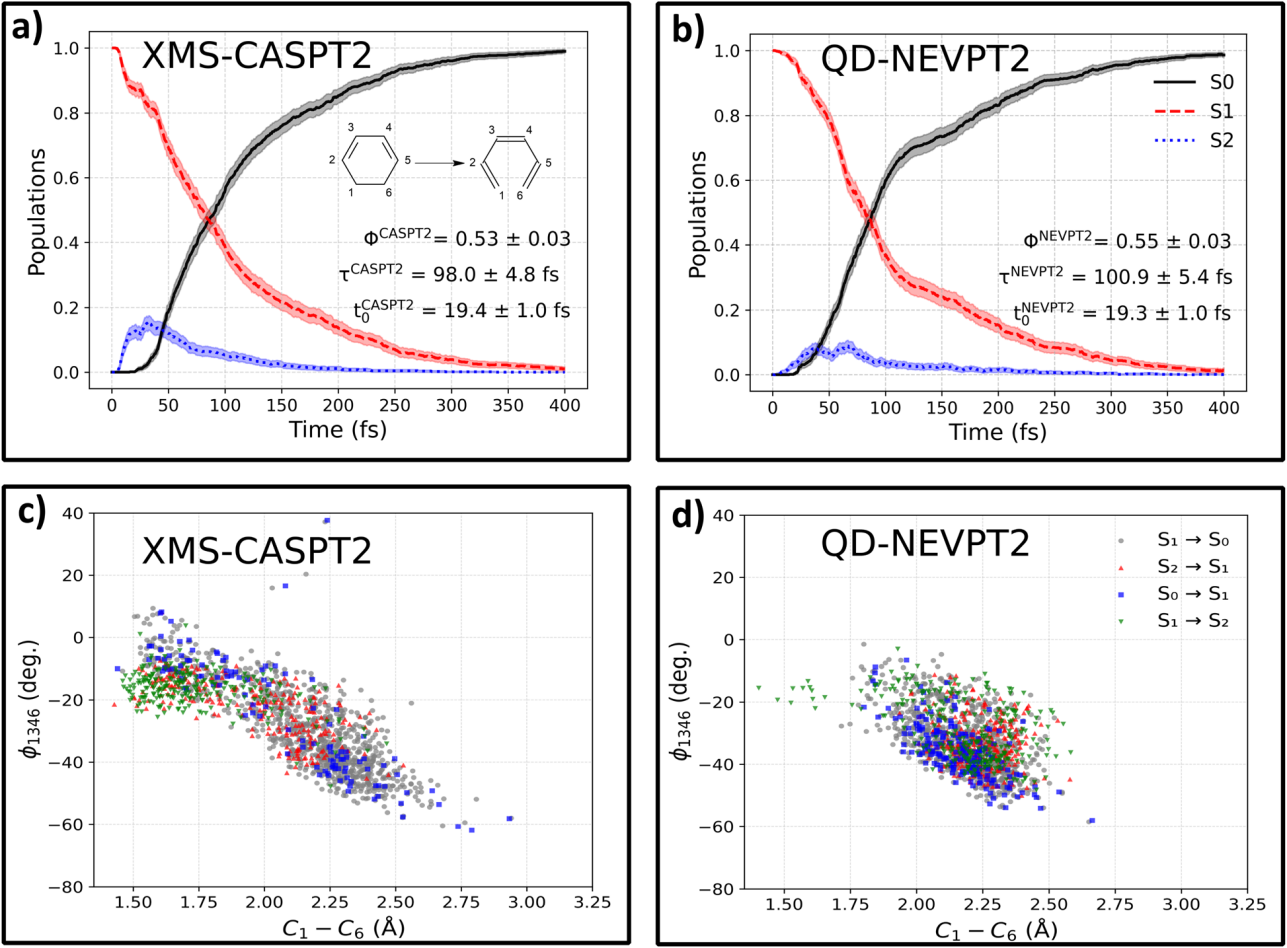
Photoinduced ring-opening dynamics of 1,3-cyclohexadiene (CHD; inset in panel (a)) following the S_0_ → S_1_ excitation, comparing gradient-free ML-NAMD simulations at the ML@XMS-CASPT2 (left column, panels (a) and (c)) and ML@QD-NEVPT2 (right column, panels (b) and (d)) levels of theory. Panels (a) and (b) show electronic-state population evolution. Both methods predict similar deactivation timescales (*τ* ≈ 100 fs) and quantum yields (*Φ* ≈ 0.53), but differ in S_2_ state involvement: XMS-CASPT2 shows higher S_2_ occupancy (∼15% maximum) with a single broad peak, while QD-NEVPT2 exhibits lower S_2_ participation (∼9% maximum) with two peaks. Panels (c) and (d) display correlation plots between the C_1_–C_3_–C_4_–C_6_ dihedral angle (*ϕ*_1346_) and the C_1_–C_6_ bond distance at S_1_ ↔ S_0_, and S_2_ ↔ S_1_ hopping geometries.

To test our approach on a more complex photochemical process, we simulate the photoinduced ring-opening dynamics of 1,3-cyclohexadiene (CHD). It is one of the most prototypical photochemical processes, with similar photoswitchable moieties found in biologically active systems^53^ and diarylethene photoswitches,^54^ among others. After optical excitation, CHD can undergo a pericyclic ring opening reaction, forming hexatriene (HT), with a schematic representation of this reaction presented in the inset of Fig. 3, panel (a). For training our ML models, we use data sourced from the SHNITSEL (Surface Hopping Nested Instances Training Set for Excited-state Learning) repository,^55^ which is comprised of 138 NAMD trajectories of CHD propagated at the XMS-CASPT2(6,6) level in ref. 56 by Polyak *et al.* (119826 conformations), using purely quantum chemical methods without machine learning. First, the OMNI-P2x model was fine-tuned to the target XMS-CASPT2 level of theory using energies alone (discarding the available high-level gradients), with auxiliary low-level AIQM1/MRCI data (both energies and gradients). We rigorously test whether training without high-level gradients can reproduce these reference XMS-CASPT2 dynamics and later re-label the dataset to the QD-NEVPT2 level of theory, together with auxiliary AIQM1/MRCI data, and compare the theoretical description provided by the XMS-CASPT2 and QD-NEVPT2 methods.

State-averaged electronic state population evolution at the ML@XMS-CASPT2 level is presented in panel (a) of Fig. 3. The excited-state evolution is in good agreement with the reference results, showing fast, partial population transfer to the S_2_ state within the first 50 fs and subsequent decay to the ground state. We fit the timescale of this deactivation process with a delayed exponent function of the form *P*^S_0_^(*t*) = (1 − exp(–(*t* − *t*_0_)/*τ*)), with *τ* being the deactivation timescale and *t*_0_ the lag time. The obtained results of *τ*^CASPT2^ = 98.0 ± 4.8 fs, *t*_0_^CASPT2^ = 19.4 ± 1.0 fs match the reference values (*τ* = 72 ± 9 fs, *t*_0_ = 16 ± 2 fs)^56^ closely, and the small discrepancy of *ca.* 25 fs can likely be attributed to the different surface hopping algorithms used (fewest-switches surface hopping (FSSH) in ref. 56 and Landau–Zener–Belyaev–Lebedev (LZBL) surface-hopping in the present case; see the Methods). Furthermore, we calculate the predicted quantum yield (QY) of the photoprocess as the fraction of reactive trajectories (taking the final bond length C_1_–C_6_ > 1.8 Å as a fingerprint of the HT isomer) relative to the total number of trajectories: *Φ* = *N*_reactive_/*N*_traj_ to obtain *Φ*^CASPT2^ = 0.53 ± 0.03, which agrees with the reference value of 0.48 ± 0.09.

The obtained ML@QD-NEVPT2 results stay in line with XMS-CASPT2 dynamics. The predicted timescale *τ*^NEVPT2^ = 100.9 ± 5.4 fs and lag time *t*_0_^NEVPT2^ = 19.3 ± 1.0 fs are almost equal to the XMS-CASPT2 timescale, which is also true for the quantum yield, with *Φ*^NEVPT2^ = 0.54 ± 0.03. A qualitative difference can be observed in the evolution of the S_2_ state, which is less populated at the ML@QD-NEVPT2 level of theory, reaching a maximum occupancy of 9%, compared to 15% for ML@XMS-CASPT2.

Next, we compare the correlation plots between the dihedral angles formed by atoms C_1_, C_3_, C_4_ and C_6_ and the distance between the two bond breaking carbon atoms, C_1_–C_6_, at hopping points, as shown in panels (c) and (d) for ML@XMS-CASPT2 and ML@QD-NEVPT2. The overall shape of the correlation plot is very similar to the reference XMS-CASPT2 results, with a clear tendency for S_2_ → S_1_ and S_1_ → S_2_ hopping occurring at larger dihedral angles and less elongated C_1_–C_6_ bonds (closer to CHD), with averages for S_2_/*S*_1_ hoppings being 〈*ϕ*_1346_〉_S_2_/S_1__^CASPT2^ = −19.76° and 〈C_1_–C_6_〉_S_2_/S_1__^CASPT2^ = 1.85 Å, compared to 〈*ϕ*_1346_〉_S_1_/S_0__^CASPT2^ = −28.90° and 〈C_1_–C_6_〉_S_1_/S_0__^CASPT2^ = 2.14 Å for S_1_/S_0_ nonadiabatic events. This tendency is much less prevalent in QD-NEVPT2 simulations, with 〈*ϕ*_1346_〉_S_2_/S_1__^NEVPT2^ = −31.43° and 〈C_1_–C_6_〉_S_2_/S_1__^NEVPT2^ = 2.21 Å, being almost equal to 〈*ϕ*_1346_〉_S_1_/S_0__^NEVPT2^ = −34.03° and 〈C_1_–C_6_〉_S_1_/S_0__^NEVPT2^ = 2.19 Å. This indicates that at the QD-NEVPT2 level of theory, the S_2_/S_1_ conical intersection lies closer to the S_1_/S_0_ crossing point, which also explains the lower overall population of the S_2_ state. Indeed, optimizing the S_2_/S_1_ minimum energy conical intersection (MECI) with both ML models yields a C_1_–C^MECI/CASPT2^_6_ distance of 2.18 Å, while for QD-NEVPT2 the optimization converges to C_1_–C^MECI/NEVPT2^_6_ = 2.27 Å. A more in-depth discussion about the differences between CASPT2 and NEVPT2, as well as linearly interpolated reaction paths at both levels of theory, can be found in the SI, Section S2.

### Simulation of *trans* → *cis* photoisomerisation of azobenzene

Having validated target-gradient-free ML-NAMD on benchmark systems, we now tackle a long-standing challenge in computational photochemistry: fully dimensional simulations of *trans*-azobenzene photoisomerization with high-level multireference methods. This photoprocess is particularly challenging for traditional QM/FSSH approaches, as not only is azobenzene prohibitively large for propagating NAMD with highly-accurate quantum chemical methods, but also the timescale for the *trans*-azobenzene (TAB) → *cis*-azobenzene (CAB) reaction is relatively large, with experimental studies pointing to values between 2 ps^57,58^ and 16 ps,^59^ requiring propagating NAMD trajectories for many time steps.

As a consequence, computational studies of this process had to resort to a wide range of approximations to make the simulations computationally feasible. Common approaches included semi-empirical methods, such as a reparameterized AM1 Hamiltonian^41,42^ or the OM2 (ref. 43) and related ODM2 methods.^44^ While semi-empirical methods were able to reproduce the quantum yield of the photoprocess with reasonable accuracy, particularly in the case of delta-learning AIQM1,^44^ the predicted timescale of the photoprocess was too fast, with all trajectories relaxing to the ground-state within 1 ps. More recently, Martinez *et al.*^2^ used the hole–hole Tamm–Dancoff approximation density functional theory with ab inito multiple spawning (AIMS) to study this photoprocess, obtaining a timescale of 6 ps. Another common approach of increasing the affordability of such simulations is reducing the dimensionality of the PES: as done in ref. 45 by using a RATTLE algorithm to freeze C–H bonds, or in ref. 46 by constructing a three-dimensional PES composed of the two C–N=N angles and the C–N=N–C dihedral at a high RASPT2 level of theory. Other approaches involved the use of TD-DFT with forced jumps at low energy gaps (due to the failure of TD-DFT at describing conical intersections)^60^ or a modified force-field method with an energy-gap law.^61^ In most cases, computational feasibility required compromising either the level of theory, the dimensionality, or the dynamics method itself. Here, we demonstrate that the proposed cutting-edge ML protocol enables ML-NAMD simulations that not only become feasible thanks to the substantial reduction in computational cost provided by ML methods, but can also be performed at levels of theory inaccessible to conventional QM approaches lacking analytic energy gradients. This strategy removes the need to compromise between using fully dimensional PESs and high-level theoretical methods, allowing the application of the latest QM developments even when analytical gradient expressions are unavailable.

**Fig. 4:**
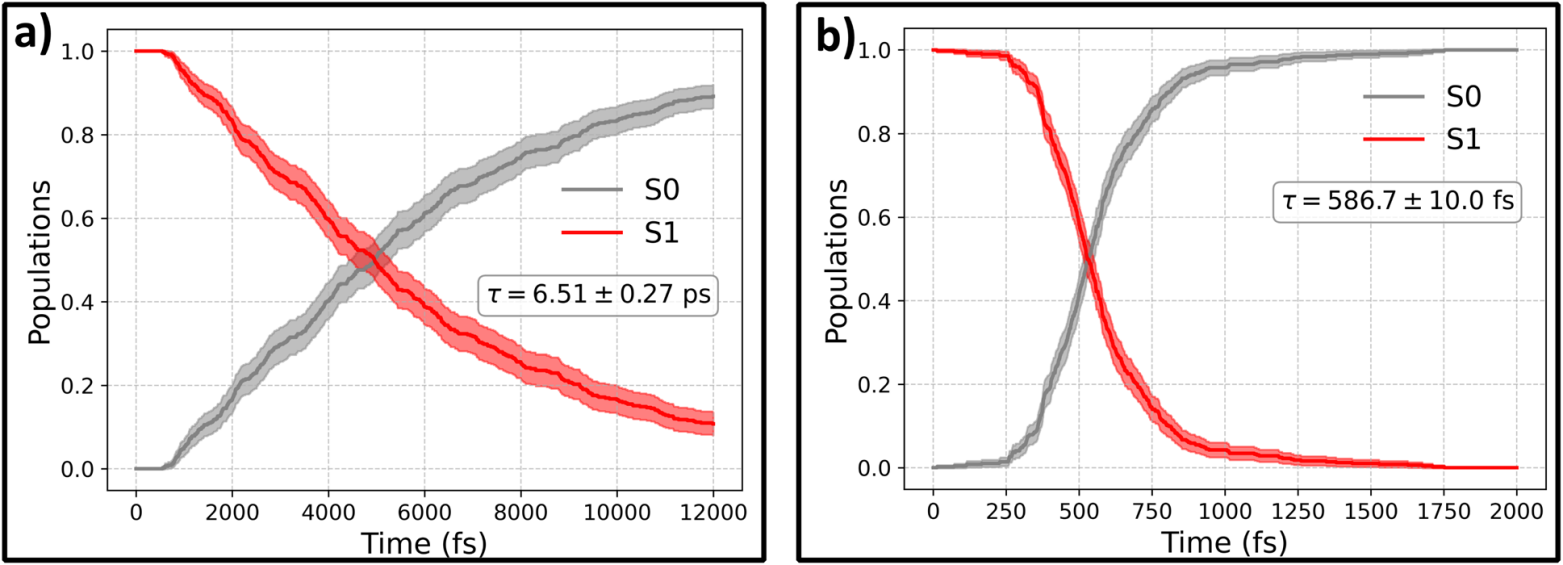
Electronic state population evolution during *trans*-azobenzene photoisomerization following the S_0_ → S_1_ (*n*π*) excitation, comparing gradient-free ML-NAMD at two levels of theory. Panel (a) shows ML@CASSCF(10,10) dynamics with a deactivation timescale of *τ* = 6.51 ± 0.27 ps. Panel (b) shows ML@QD-NEVPT2(10,10) dynamics with a significantly faster timescale of *τ* = 586.7 ± 10.0 fs. Red lines indicate S_1_ population decay; black lines show ground-state (S_0_) recovery. Timescales were obtained by fitting *P*(*t*) = 1 − exp(−*t*/*τ*) to the S_0_ population. Shaded regions represent 95% confidence intervals from 500 trajectories.

The evolution of the excited-state population at two levels of theory: ML@CASSCF (10,10) and ML@QD-NEVPT2 (10,10) is presented in Fig. 4. ML models used to propagate the dynamics were trained on a dataset taken from ref. 29, collected using AL at the AIQM1/MRCI level of theory. Due to the versatality of the AIO approach, we trained a single model on three levels of theory: target CASSCF(10,10) and QD-NEVPT2(10,10), with no gradient labels, as well as auxiliary AIQM1/MRCI data with energies and gradients, with more details provided in the Methods.

The timescales obtained with both these methods differ significantly, with predicted time-constants for S_1_ → S_0_ deactivation being equal to 6.51 ± 0.27 ps for ML@CASSCF and 586.7 ± 10.0 fs for ML@QD-NEVPT2. Time constants were obtained by fitting an exponential function *P*(*t*) = (1 − exp(−*t*/*τ*)) to the S_0_ populations, with uncertainties estimated by one standard error obtained from bootstrapping with 1000 samples.

Surprisingly, it is the CASSCF results that agree much better with experimental studies. The agreement can be further quantified by looking at the predicted quantum yields of the photoisomerization processes, which are calculated as the fraction of the trajectories relaxing to form the CAB isomer, *N*_reactive_, relative to the total number of trajectories *N*_traj_: *Φ* = *N*_reactive_/*N*_traj_, with CAB defined as having a dihedral angle less than 60°. The quantum yield obtained from the CASSCF simulations is *Φ*_CASSCF_ = 0.22 ± 0.04 and from QD-NEVPT2 dynamics *Φ*_NEVPT2_ = 0.45 ± 0.04. Again, the ML@CASSCF results are much closer to experimental measurements, which typically report a quantum yield between 0.2 and 0.3, after nπ* (S_1_) excitation. On the other hand, the higher QY predicted by ML@QD-NEVPT2 simulations agrees well with the result presented in ref. 46 obtained at the RASPT2 level of theory: 0.44. While this outcome may be surprising, it is consistent with numerous literature examples of CASSCF-based NAMD propagation yielding correct results.^62–64^ We believe that this can be primarily attributed to a favorable cancellation of errors between the trajectory surface hopping approximation and the quality of the CASSCF PES. In contrast, while QD-NEVPT2 is indeed a more theoretically-rigorous method that accounts for both the static and dynamic electronic correlation (contrary to CASSCF, which includes only the static part), this method has not yet been tested for NAMD propagation, and this works shows the first example of such an application.

**Fig. 5:**
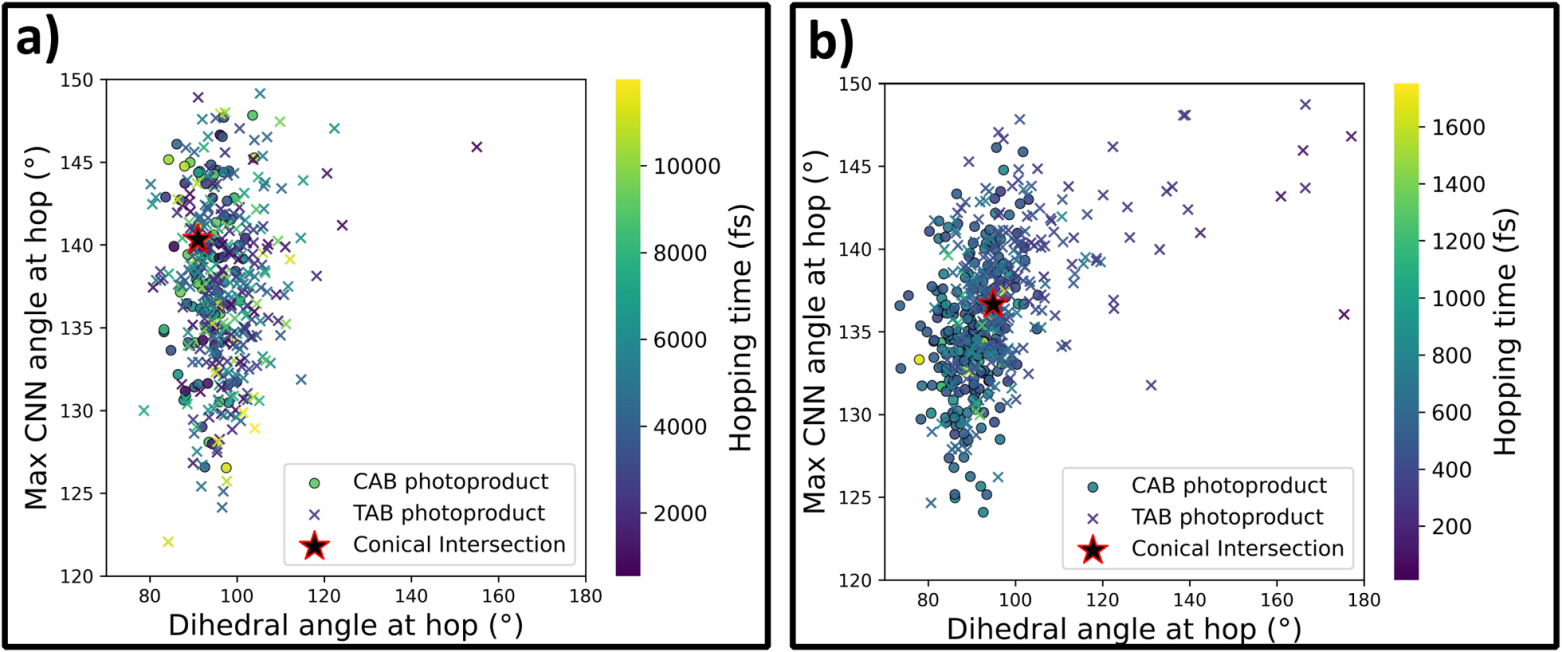
Correlation between key molecular coordinates at S_1_ → S_0_ hopping geometries during *trans*-azobenzene photoisomerization. Panel (a) shows ML@CASSCF(10,10) dynamics, panel (b) shows ML@QD-NEVPT2(10,10) dynamics. The *x*-axis represents the C–N=N–C dihedral angle and the *y*-axis shows the maximum C–N=N angle. Points are color-coded by hopping time, with circles indicating trajectories that form *cis*-azobenzene (CAB) and crosses indicating trajectories that retain *trans*-azobenzene (TAB). Stars mark the S_1_/S_0_ MECIs optimized at each level of theory. Most hops occur near the respective MECIs, with CAB formation being more likely near the conical intersection and TAB preferentially formed through earlier deactivation at more planar geometries.

**Fig. 6:**
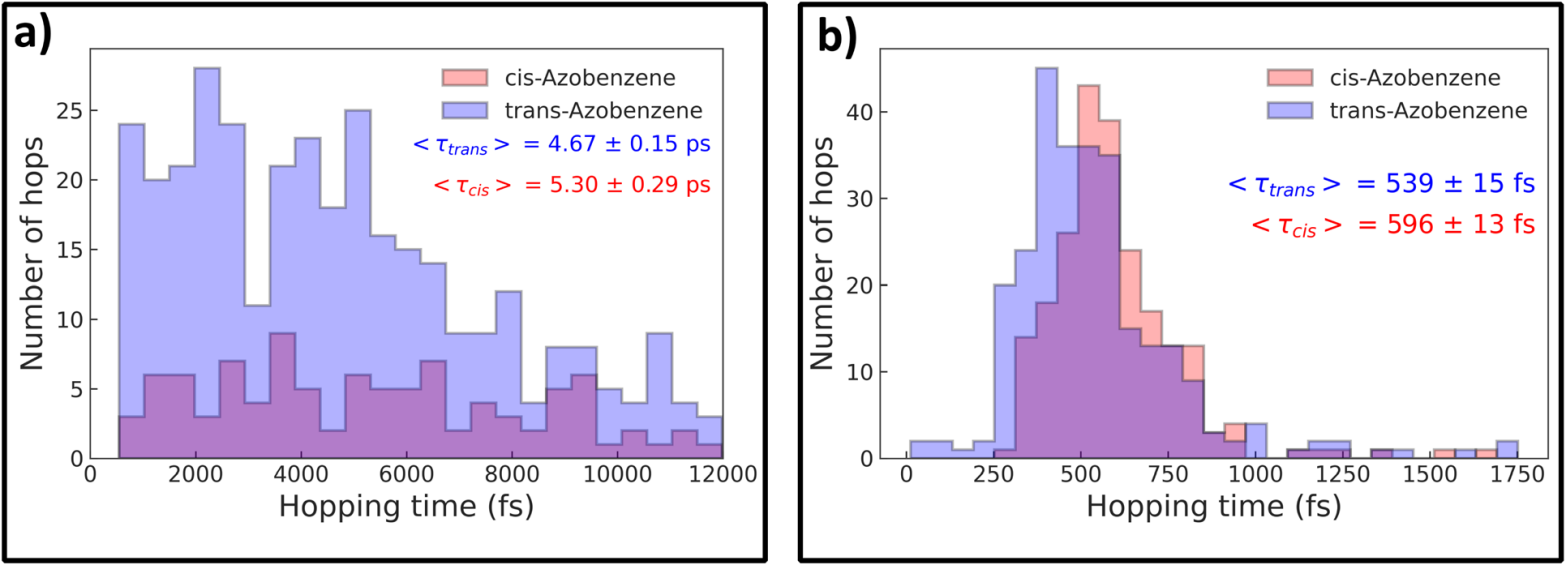
Distribution of S_1_ → S_0_ hopping times during *trans*-azobenzene photoisomerization for ML@CASSCF(10,10) (a) and ML@QD-NEVPT2(10,10) (b) dynamics. Red bars indicate trajectories forming *cis*-azobenzene (CAB); blue bars indicate trajectories retaining *trans*-azobenzene (TAB). Average hopping times (〈τ〉) are shown for each photoproduct. TAB formation occurs earlier than CAB formation at both levels of theory, consistent with the nonproductive decay mechanism through early deactivation at planar geometries.

As a final step in this investigation, we can take a closer look at the S_1_ → S_0_ deactivation points in both sets of trajectories, which are plotted in Fig. 5 as a correlation plot between the two key molecular coordinates relevant for this photoprocess: the C–N=N–C dihedral angle and the maximum C–N=N angle. Crosses represent nonadiabatic events that led to the formation of the TAB photoproduct, while circles represent CAB. For both the methods, most of the hopping occurs in the direct vicinity of the conical intersection, as optimized at the respective level of theory (details provided in the Methods section). At the same time, a clear tendency for the preference of TAB formation is seen for hops occurring away from the CI, with more planar structures and earlier hop times. This agrees with the results of Weingart *et al.*,^65^ who predicted the possibility of premature, nonproductive decay to the ground-state in this process. This mechanism is more pronounced at the ML@QD-NEVPT2 level of theory, with 8% of the trajectories deactivating outside the direct vicinity of the conical intersection (defined as having the dihedral angle within ±20° of the CI), while this mechanism occurs only in 3% of the CASSCF trajectories. In both cases, these trajectories form exclusively the TAB isomer. Furthermore, in Fig. 6 we can see the distribution of hopping times leading to the formation of both isomers. In both cases, the average time of TAB formation is significantly lower than CAB, further evidencing the mechanism of early hopping favoring the nonproductive decay. The simulations allow us to determine the branching ratio of CAB : TAB at the conical intersection, *Γ*, defined as the ratio of trajectories relaxing to each photoproduct in the immediate vicinity of the CI. We obtain *Γ*_CASSCF_ = 1 : 3.32 and *Γ*_NEVPT2_ = 1 : 1.05. The difference between these values—likely arising from variations in the conical intersection structure—can be attributed to the discrepancy in the predicted quantum yields of the photoprocess at the two levels of theory, while mechanistic differences play a less relevant, secondary role.

## Discussion

In this work, we present a procedure that allows performing nonadiabatic molecular dynamics (NAMD) simulations without requiring access to analytical energy gradients from the underlying quantum chemical method. Our approach relies on fine-tuning a preexisting foundational excited-state machine learning potential (OMNI-P2x^40^ in the present work) to reproduce the relevant potential-energy surfaces and exploiting its differentiability to obtain the forces necessary for NAMD propagation, which was tested at multiple levels of theory: AIQM1/MRCI, CASSCF, MRSF-TD-DFT and QD-NEVTP2. This strategy not only reduces the cost of generating training data but also enables dynamics simulations at levels of theory for which analytical gradients are unavailable.

We validate the protocol on the benchmark system fulvene, demonstrating that gradient-free ML models can accurately reproduce NAMD dynamics for three reference methods: AIQM1/MRCI, CASSCF, and MRSF-TDDFT. The method is then extended to the QD-NEVPT2 level of theory, where reference gradients are inaccessible, allowing, for the first time, NAMD propagation at this level of theory. We then show that the proposed protocol can work in a more complex simulation: the photoinduced ring opening dynamics of cyclohexadiene. Comparison of the results obtained with an ML model trained without access to XMS-CASPT2 gradients yields excellent agreement between ML-NAMD and reference quantum-chemical calculations. Comparison with ML@QD-NEVPT2 dynamics reveals a consistent description of the photoreaction, with a lesser role of the S_2_ state.

Finally, we apply our methodology to perform fully dimensional simulations of *trans*-azobenzene photoisomerization at both the CASSCF and QD-NEVPT2 levels. The obtained dynamics are compared with previous theoretical and experimental studies, establishing the present results as the highest-level, fully dimensional simulations of this photoprocess performed to date. Overall, this work demonstrates that target-gradient-free ML-based NAMD can unlock access to highly accurate electronic-structure methods previously unusable for dynamics, paving the way toward routine simulations at the frontier of excited-state theory.

## Methods

### Fulvene

CASSCF calculations used as a reference method for TSH simulations were performed through the interface to the COLUMBUS quantum chemistry package,^66^ with an active space of 6 electrons in 6 orbitals, using the Dunning correlation-consistent double-ζ basis set, cc-pVDZ.^67^ QD-NEVPT2 calculations were performed in ORCA 5.0.3,^68,69^ with the same active space and basis set as CASSCF.

The semi-empirical part of AIQM1/MRCI-SD calculations was performed using the half-electron restricted open-shell Hartree–Fock formalism in the SCF procedure, with HOMOs and LUMOs singly occupied. This configuration was then supplemented with two additional references: a closed-shell HOMO–HOMO configuration and a doubly-excited LUMO–LUMO configuration. The active space spanned 6 electrons in 6 orbitals. The MRCI wavefunction was constructed by allowing single and double excitations within a such-defined active space.

Mixed-reference spin flip TD-DFT calculations were performed in the OpenQP software package, version 1.0, using the Becke–Lee–Yang–Parr “half-and-half” functional (BH&H-LYP) and the def2-SVP basis set.^70^

Trajectory surface hopping was propagated using the coupling-free LZBL formalism, as implemented in MLatom,^44^ with hopping probabilities between states *k* and *j*, *P*_*j*→*k*_ calculated using the following formula:1
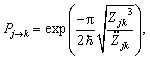
where *Z*_*jk*_ is the energy gap between states *j* and *k*, and *Z̈*_*jk*_ is its second-order time derivative, with the hopping probability evaluated only at local minima of the energy gap. Recent benchmark studies show that this algorithm can provide accurate results, compared with Tully's fewest-switches surface hopping algorithm.^44,71^ 1000 initial conditions were selected *via* Wigner sampling, and all trajectories were initialized in the first excited state, with two active states, S_1_ and S_0_. A time step of 0.1 fs was used, along with the reduced kinetic energy reservoir setting, with trajectories propagated for 60 fs. For CASSCF, a total of 623 trajectories were used as reference, with the same time step and propagation time. Mixed-reference spin-flip TD-DFT calculations were performed with the OpenQP software package (version 1.0)^10,11,72,73^ interfaced to MLatom, using the Becke–Lee–Yang–Parr half-and-half exchange–correlation functional (BH&H-LYP)^74^ and the def2-SVP basis set.^70^

Due to the substantial computational cost of MRSF-TDDFT, the integration time step was increased to 0.5 fs for both the reference and ML-propagated dynamics; this change did not affect total-energy conservation. The OpenQP trajectories exhibited limited numerical stability, primarily caused by self-consistent-field convergence failures in certain geometries. Out of 400 propagated trajectories, 220 (55%) completed successfully. A separate test set of 100 trajectories using a 0.1 fs time step achieved a similar success rate (43%), indicating that the stability issues originate from the electronic-structure calculations rather than the integration parameters. Consequently, population statistics derived from these reference trajectories should be interpreted with caution.

The statistical uncertainties of the populations arising from a finite number of propagated trajectories, Δ*P*(*t*), were estimated using the normal approximation interval for a binomial process which, for a confidence interval of 95%, takes the form:2
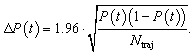


ML-NAMD was driven using the OMNI-P2x model, after fine tuning for each set of dynamics, for all cases presented in this work. A single model from the ensemble of three OMNI-P2x energy MLIPs was fine-tuned. In the cases of AIQM1/MRCI, MRSF-TDDFT and QD-NEVPT2, the training was performed simultaneously on the target level of theory (without gradients included) and on the CASSCF data (with gradients included). Individual levels of theory were one-hot-encoded, with more details on the OMNI-P2x architecture and fine-tuning available in ref. 40.

### Cyclohexadiene

XMS-CASPT2 calculations used for labeling the CHD dataset (R02 in the SCHINTSEL repository) were performed with the BAGEL program,^75^ with an active space of 6 electrons in 6 orbitals, within the cc-pVDZ basis set. More details about the electronic structure settings used are available in ref. 56. QD-NEVPT2 calculations were performed with the same settings using the ORCA quantum chemistry package. The final number of labeled datapoints was 119826 for XMS-CASPT2 and 106414 for QD-NEVPT2, with losses due to CASSCF convergence.

ML model training was performed at the target level of theory (XMS-CASPT2 or QD-NEVPT2), labeled with energies only, and included semi-empirical AIQM1 labels containing energies and forces. The inclusion of low-level forces was found to be crucial for trajectory stability, decreasing the number of unphysically dissociated trajectories from about 80% to 2% in the case of XMS-CASPT2.

TSH propagation was performed using the same algorithm as for fulvene, with the reduced kinetic energy reservoir setting turned off. The maximum propagation time was set to 400 fs, with a 0.5 fs time step. A total of 1000 trajectories were propagated at each level of theory, with 21 (2.1%) trajectories removed from final analysis at the XMS-CASPT2 level of theory, and 64 (6.4%) trajectories removed at the QD-NEVPT2 level of theory, due to reaching unphysical, highly distorted geometries.

Conical intersections presented in this work have been optimized using the penalty function algorithm CIopt,^76^ using the ML-predicted energies and gradients, as implemented in MLatom 3.19.1, with an interface to the geomeTRIC package.^77^

### Azobenzene

The training set from ref. 29 was used for fine-tuning the OMNI-P2x model, with details of the data collection process presented therein. Data labeling was performed in ORCA 5.0.3, with CASSCF and subsequent QD-NEVPT2 calculations using the cc-pVDZ basis set and an active space of 10 electrons in 10 orbitals, which was found to give better convergence of CASSCF calculations than the active space of 8 electrons and 10 orbitals used in ref. 2. After labeling, the final training set consisted of 24995 points with QD-NEVPT2 and CASSCF energies.

The ML model was trained simultaneously on all 3 levels of theory available (AIQM1/MRCI, CASSCF, and QD-NEVPT2). As OMNI-P2x was trained using two levels of theory, three additional input neurons were initialized in the NN, fully connected to the first hidden layer. The AIQM1/MRCI, CASSCF and QD-NEVPT2 levels of theory were one-hot-encoded with the following descriptors: [0,0,1,0,0], [0,0,0,1,0] and [0,0,0,0,1], respectively. The predictions utilized the target level of theory descriptor. Contrary to fulvene and CHD, where one model was used for dynamics propagation, an ensemble of four models with the same initial weights was trained, not only to provide better stability and accuracy of the resulting dynamics, but also to provide uncertainty quantification of their propagation. The ensemble average of energy and energy gradients was used for propagation. The uncertainty was evaluated as the standard deviation between the predictions of the four models at the target level of theory.

500 trajectories were propagated for CASSCF and QD-NEVPT2, with the same surface hopping propagation scheme as used in the fulvene dynamics, with a time step of 0.5 fs, for a total time of 3 ps for QD-NEVPT2 dynamics and 12 ps for CASSCF dynamics. Initial conditions were selected by filtering to an excitation window corresponding to the S_0_ → S_1_ absorption maximum, with details provided in ref. 29. We judge the predictions as confident, as only 7 trajectories (1%) exceeded a commonly-used threshold of 0.03 hartree^8,62^ before deactivating to the ground-state in the case of QD-NEVPT2 dynamics. These trajectories were removed from further analysis. In the case of CASSCF dynamics, 16 (3%) highly uncertain, distorted trajectories were removed from the analysis.

### Training and inference

MLIP training was performed using an initial learning rate of 0.001, with a reduction on plateau LR scheduler with a patience of 50 epochs and a factor of 0.5. Training was carried out until the LR dropped below 10^−5^. A batch size of 16 was used for fulvene and 256 for CHD and azobenzene, depending on the size of the training set. A loss function *L*, containing energy, energy gradient and gap terms was used, as defined in ref. 29:

3 *L* = *ω*_E_*L*_E_ + *ω*_F_*L*_F_ + *ω*_gap_*L*_gap_,

with ‖*E*^ML^ − *E*^ref^‖^2^ and ‖*F*^ML^ + *F*^ref^‖^2^. The default *ω* loss coefficients in the MLatom software^44,78^ are 1, 1, and 0.1, for energies, gaps, and energy gradients, respectively. We used them for fulvene, while for CHD and azobenzene we lowered the gap coefficient to 0.1, in order to balance the magnitude of different terms of the loss function. Computational timings of model training and inference for all studied systems are available in Section S3 of the SI.

## Conflicts of interest

There are no conflicts to declare.

## Author contributions

M. M. wrote the original manuscript draft, designed the numerical experiments and performed them, as well as developed the necessary code, collected and labeled the training data, analyzed and visualized the results. J. J. contributed to the result analysis and interpretation, co-supervised research, and secured funding. H. L. co-designed and co-conceived the project, as well as introduced the idea of gradient-free learning. P. O. D. supervised the project and co-designed the experiments and simulations performed. All authors discussed the results and revised the manuscript.

## Supplementary Material

SC-017-D5SC09557C-s001

## Data Availability

The data supporting presented results (training sets and ML models) are available at https://doi.org/10.6084/m9.figshare.30774632, under the MIT license. Tutorials for the presented methods will be made available at: https://aitomistic.com/mlatom/tutorial_omnip2x.html. Supplementary information (SI): additional evaluations and computational timings. See DOI: https://doi.org/10.1039/d5sc09557c.
