# Supplementary material for: Gradients not needed: ML-driven propagation of nonadiabatic molecular dynamics without reference gradients
Source: Chem Sci. 2026 Jan 22;17(12):6043–54. doi: 10.1039/d5sc09557c (PMC12857564; doi:10.1039/d5sc09557c)
Supplement: SC-017-D5SC09557C-s001 [file SC-017-D5SC09557C-s001.pdf]

Supplementary Information (SI) for Chemical Science.
This journal is © The Royal Society of Chemistry 2026

Supporting Information for "Gradients not needed: ML-driven propagation of nonadiabatic molecular dynamics without reference gradients"

Mikolaj Martyka[1], Joanna Jankowska[⋆,1], Hans Lischka[2], and Pavlo O. Dral[⋆,3,4,5]

[1]University of Warsaw, Faculty of Chemistry, Pasteura 1, Warsaw, 02-093, Poland
[2]Department of Chemistry and Biochemistry, Texas Tech University, Lubbock, TX, USA
[3]State Key Laboratory of Physical Chemistry of Solid Surfaces, College of Chemistry and Chemical Engineering, and Fujian Provincial Key Laboratory of Theoretical and Computational Chemistry, Xiamen University, Xiamen, Fujian 361005, China
[4]Institute of Physics, Faculty of Physics, Astronomy, and Informatics, Nicolaus Copernicus University in Toruń, ul. Grudziadzka 5, 87-100 Toruń, Poland
[5]Aitomistic, Shenzhen 518000, China

Contents

S1 Effect of Incomplete Reference Data on MRSF-TDDFT Population Uncertainty S2

S2 CHD dynamics S3

S3 Computational timings S6

S3.1 Fulvene S6

S3.2 Cyclohexadiene S7

S3.3 Azobenzene S7

References S7

S1 Effect of Incomplete Reference Data on MRSF-TDDFT Population Uncertainty

To test the effect of the compromised data quality on the agreement between the S_0 rise curves, we re-compute the reference MRSF-TDDFT error bars using bootstrapping with resampling reduced to 55% of trajectories per iteration to account for the incomplete data. This provides a lower bound on the uncertainty related to missing reference data. Comparison of the population evolution with the new error bars is presented in Figure S1.

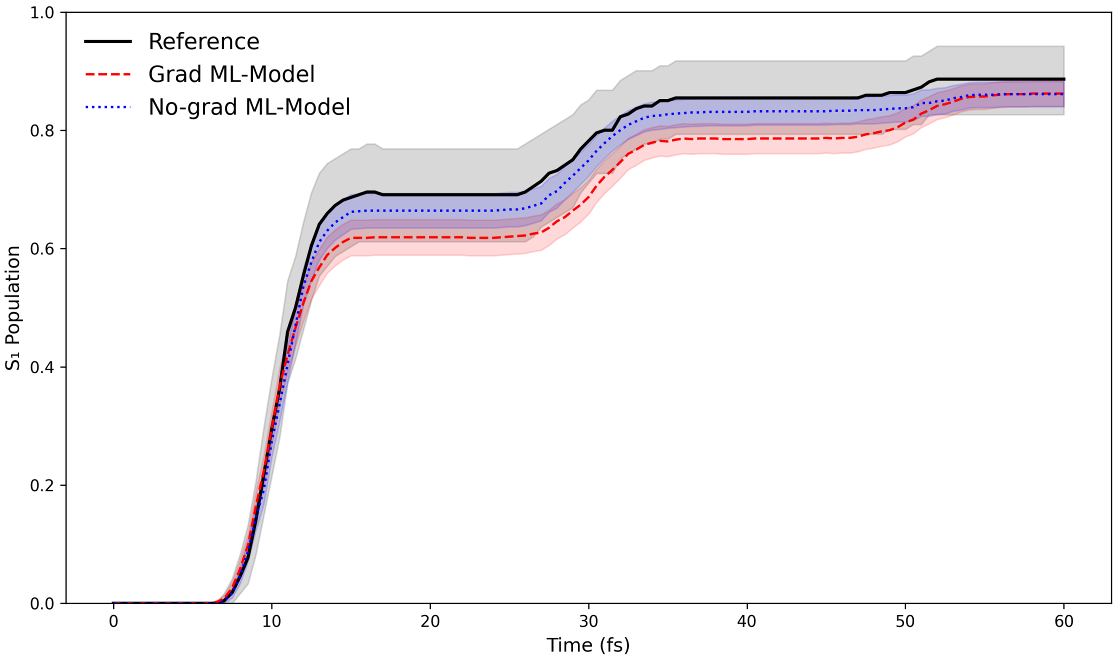

Figure S1: Ground-state population recovery in fulvene following $S_0 \rightarrow S_1$ excitation, at the MRSF-TDDFT level of theory. Black solid lines show reference quantum-chemical dynamics, red dashed lines show the ML model trained with energies and gradients, and blue dotted lines show the gradient-free model trained on energies alone. The shaded error bars for the reference calculations were computed using bootstrapping with resampling reduced to 55% of trajectories per iteration to account for the incomplete data.

With this adjustment, both the ML models fall within the 95% confidence interval error bar of the reference population. Importantly, this represents a lower bound on uncertainty, as it assumes

failures are random rather than systematic. As the failures may correspond to a particular set of geometries encountered in the dynamics, the actual error bars may be even more inflated.

S2 CHD dynamics

To both provide a benchmark for the ML models, as well as a basis for a mechanistic explanation of the reduced involvement of QD-NEVPT2, we compute linearly interpolated reaction paths between the ground-state of CHD, and the S_2/S_1 MECI, which is connected to the S_1/S_0 MECI in the second part of the reaction path, according to the proposed reaction mechanism in Ref. [5] Results are provided below, taking the XMS-CASPT2 structures of the relevant points from the supporting information of Ref. [5]. The results are computed both with reference methods (full dots), and the ML models (crosses). Results are presented below, with XMS-CASPT2 energies in panel a), and QD-NEVPT2 results in panel b).

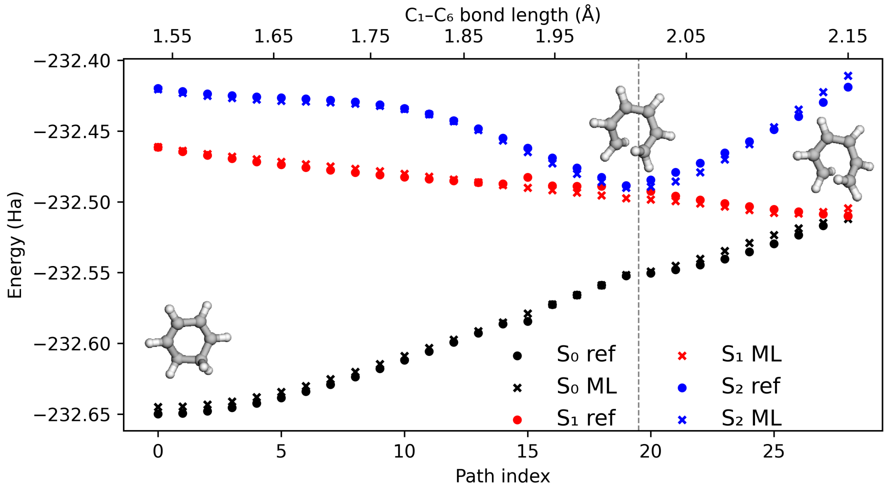

(a)

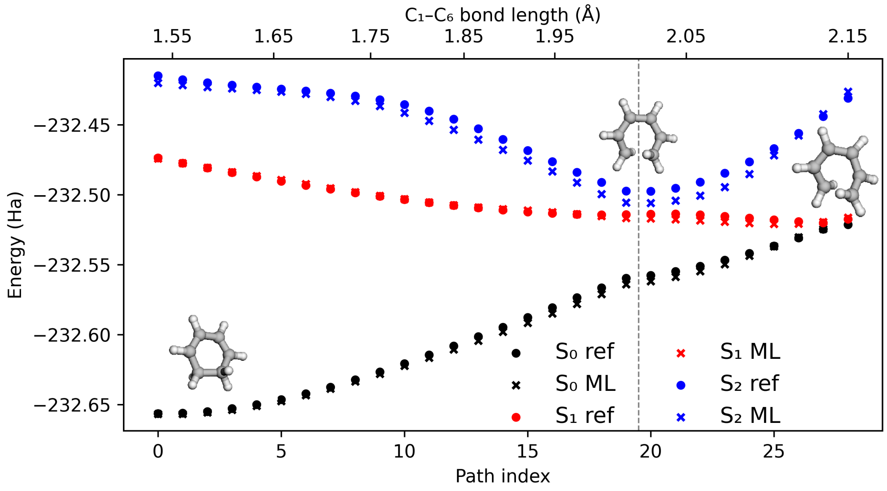

(b)

Figure S2: Linearly interpolated reaction paths connecting the ground-state minimum, the S_2/S_1 MECI (up to the dotted line), and the S_2/S_1 MECI with the S_1/S_0 MECI (second part of the path). Panel a) shows reference XMS-CASPT2 results (full dots), and ML-model predictions (crosses), while panel b) shows the same at QD-NEVPT2 level of theory. Optimized structures at the XMS-CASPT2 level of theory were taken from Ref. [5]. Insets show molecular structures at the respective optimized geometries.

Overall, the agreement between the ML models, and reference results is very good: excellent in the case of the S_0 and S_1 states, and acceptable for the S_2 state (similar or lower to other state-of-the-art models trained on sub-sets of the SCHNITSEL repository [2], in eg. Ref. [1], with access to reference gradients). This difference in accuracy reflects the sampling method of the datapoints, which were collected from NAMD trajectories, spending the predominant portion of their propagation time in the S_1 and S_0 states. The MAE values for different properties at both levels of theory are summarised in the table below.

Table S1: MAE of ML-predicted energies and energy gaps, in eV, along the linearly interpolated reaction path based on the XMS-CASPT2 structures.

Level of theory	S_0 energy	S_1 energy	S_2 energy	S_2/S_1 gap	S_1/S_0 gap
XMS-CASPT2	0.087	0.076	0.075	0.118	0.146
QD-NEVPT2	0.061	0.036	0.156	0.054	0.096

Next, we compute a linearly interpolated reaction path using the ML@QD-NEVPT2 optimized S_0 minimum, and S_2/S_1 and S_1/S_0 MECIs. Results are presented below. Compared to the XMS-CASPT2 reaction path, we can notice that: the S_2/S_1 MECI is located much further from the S_0 minimum ($C_1 - C_6$ distance of 2.25 vs 2.03) and the S_1/S_0 MECI is closer to S_2/S_1 MECI, as well as the S_1/S_0 energy gap being considerably smaller around the S_2/S_1 MECI (about 0.5 eV vs 1.73 eV from XMS-CASPT2 predictions). Both these effects: greater spatial distance to the S_2 minimum combined with a higher proximity of the S_1/S_0 CI to the S_2/S_1 MECI, as well as a lower S_1/S_0 gap will diminish the involvement of S_2 in the dynamics.

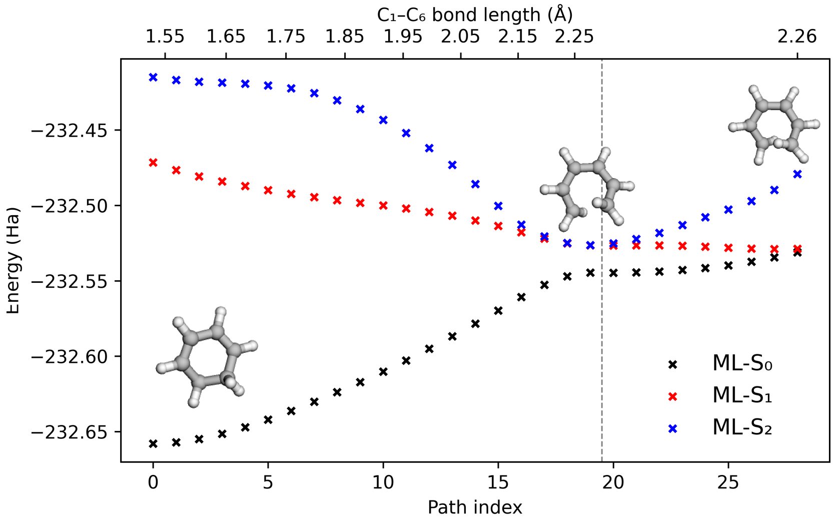

Figure S3: Linearly interpolated reaction paths connecting the ground-state minimum, the S_2/S_1 MECI (up to the dotted line), and the S_2/S_1 MECI with the S_1/S_0 MECI (second part of the path). Structures were optimized at the ML@QD-NEVPT2 level of theory, and values represent ML predictions at each level of theory. Insets show molecular structures at the respective optimized geometries.

S3 Computational timings

S3.1 Fulvene

Dataset sampling using AL at the CASSCF level of theory took about 1 day of wall-time, using a 16-core node with Intel Xeon Gold 6226R CPUs, and a single NVIDIA RTX 4090 GPU, as described in Ref. [3] of the main text.

Labeling the data took about 550 and 532.5 CPU·h at the QD-NEVPT2 and MRSF-TDDFT levels of theory, using nodes with 6 Intel(R) Xeon(R) Platinum 8268 CPU cores available. Due to the semi-empirical nature of AIQM1, the labeling cost was negligible.

The training times were between one and two hours per model, for models trained on energies and gradients, and about half an hour for models trained without reference gradients, using a single RTX 4090 GPU. The inference time on a single Intel Xeon Gold 6226R core is equal to about 10 ms for the calculation of ground and excited state energies and gradients.

S3.2 Cyclohexadiene

Re-labeling the SCHINTSEL R02 dataset to QD-NEVPT2 level of theory consumed approximately 13k CPU·h, using the same nodes with 6 Intel(R) Xeon(R) Platinum 8268 CPU cores available.
The training time (using the target-level, gradient-free dataset, and the auxiliary dataset with energy gradients) totaled to 12–16 hours per model, on the aforementioned RTX 4090.
Inference time is equal to about 14 ms per predicted point, with energies and gradients for 3 electronic states.

S3.3 Azobenzene

Collection of active learning data for azobenzene took about 3 weeks, with more details provided in Ref. [4], using the same computational setup, as for fulvene.
Data labeling at the QD-NEVPT2 level consumed about 70k CPU·h, using nodes with 12 Intel(R) Xeon(R) Platinum 8268 CPU cores.
The training time per ensemble member was about 8 hours, using the same GPU as for other systems, while the inference time of the ensemble, averaging over the predictions of all four models takes about 30 ms, for two electronic states and their gradients.

Table S2: Computational performance of the ML models for the three studied systems, in ps/day. Azobenzene propagation is significantly slower, due to the use of an ensemble of 4 ML models.

System	Fulvene	CHD	Azobenzene
Computational performance [ps/day]	1800	1550	350

References

[1] R. Barrett, C. Ortner, and J. Westermayr. Transferable machine learning potential x-mace for excited states using integrated deepsets. *arXiv preprint arXiv:2502.12870*, 2025. Accessed: 05-12-2025.

[2] R. Curth, T. E. Röhrkasten, C. Müller, and J. Westermayr. Surface hopping nested instances training set for excited-state learning. *Scientific Data*, 12(1), July 2025. ISSN 2052-4463. doi: 10.1038/s41597-025-05443-5. URL `http://dx.doi.org/10.1038/s41597-025-05443-5`.

[3] M. Martyka, X.-Y. Tong, J. Jankowska, and P. O. Dral. Omni-p2x: A universal neural network

potential for excited-state simulations. *ChemRxiv*, Apr. 2025. doi: 10.26434/chemrxiv-2025-j207x. URL http://dx.doi.org/10.26434/chemrxiv-2025-j207x. Accessed: 05-12-2025.

[4] M. Martyka, L. Zhang, F. Ge, Y.-F. Hou, J. Jankowska, M. Barbatti, and P. O. Dral. Charting electronic-state manifolds across molecules with multi-state learning and gap-driven dynamics via efficient and robust active learning. *npj Computational Materials*, 11(1), May 2025. ISSN 2057-3960. doi: 10.1038/s41524-025-01636-z. URL http://dx.doi.org/10.1038/s41524-025-01636-z.

[5] I. Polyak, L. Hutton, R. Crespo-Otero, M. Barbatti, and P. J. Knowles. Ultrafast photoinduced dynamics of 1, 3-cyclohexadiene using xms-caspt2 surface hopping. *Journal of Chemical Theory and Computation*, 15(7):3929–3940, June 2019. ISSN 1549-9626. doi: 10.1021/acs.jctc.9b00396. URL http://dx.doi.org/10.1021/acs.jctc.9b00396.
